# Supplementary figures and images for: Predictive modeling of Pseudomonas syringae virulence on bean using gradient boosted decision trees
Source: PLoS Pathog. 2022 Jul 25;18(7):e1010716. doi: 10.1371/journal.ppat.1010716 (PMC9352200; doi:10.1371/journal.ppat.1010716)

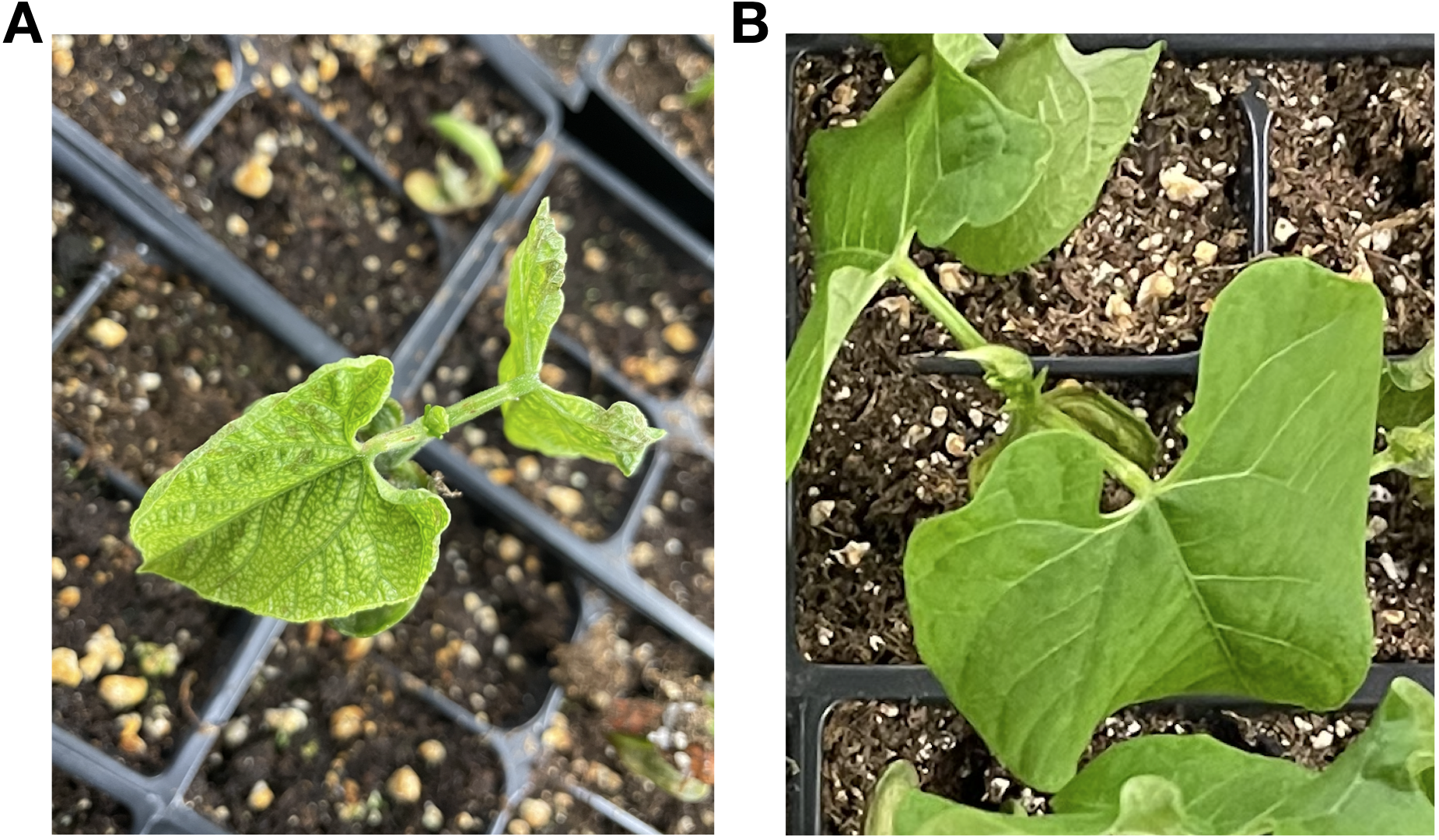

Supplement: S1 Fig — Halo blight leaf symptoms on a plant infected with Phh1448A on the left, compared to healthy plants treated with MgSO4 on the right. (TIF) [file ppat.1010716.s001.tif]

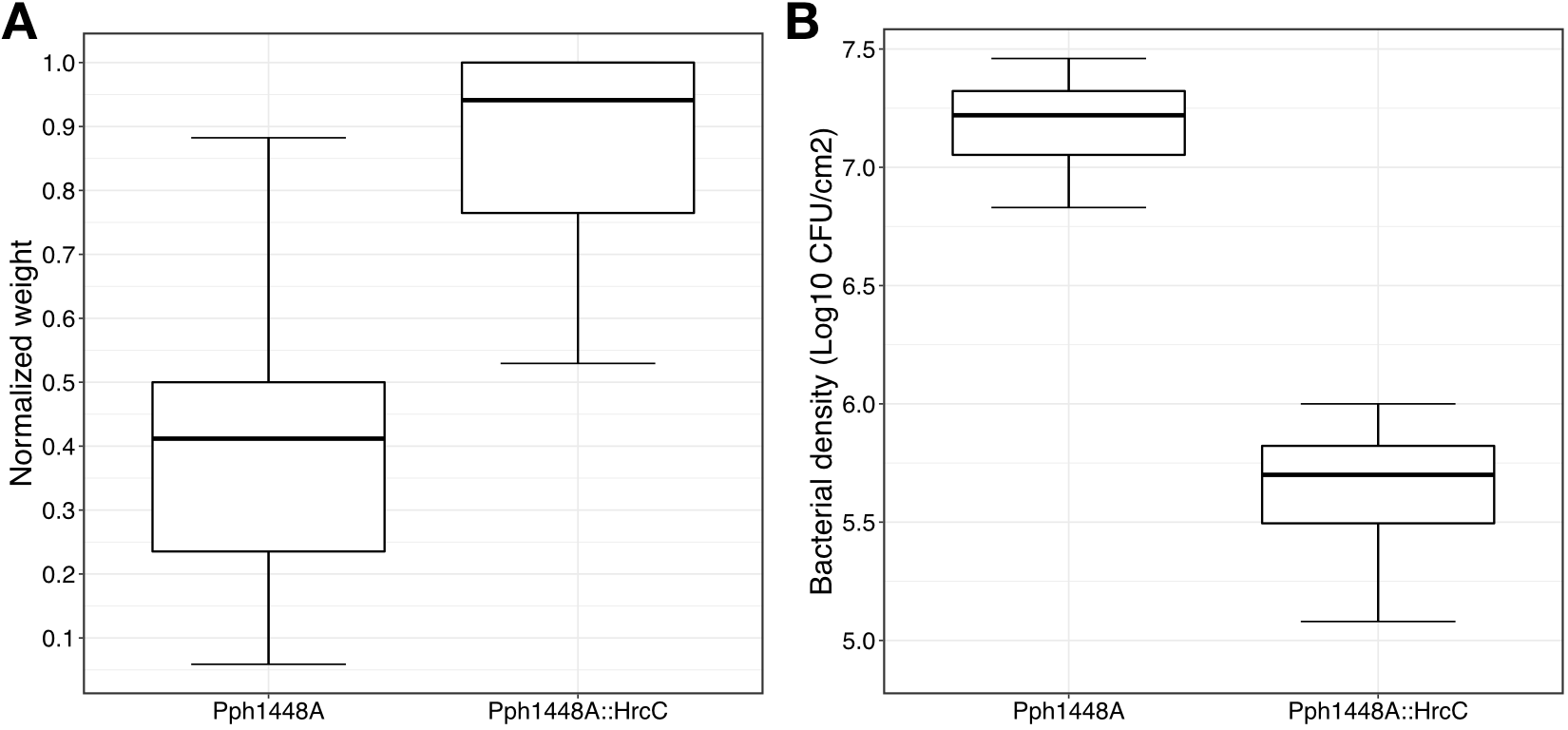

Supplement: S2 Fig — The mutant Pph1448A::HrcC is unable to deliver T3SEs into the host cell. Plants treated with this mutant therefore exhibit higher normalized fresh weights and lower bacterial densities in comparison to a wild-type treatment. (TIF) [file ppat.1010716.s002.tif]

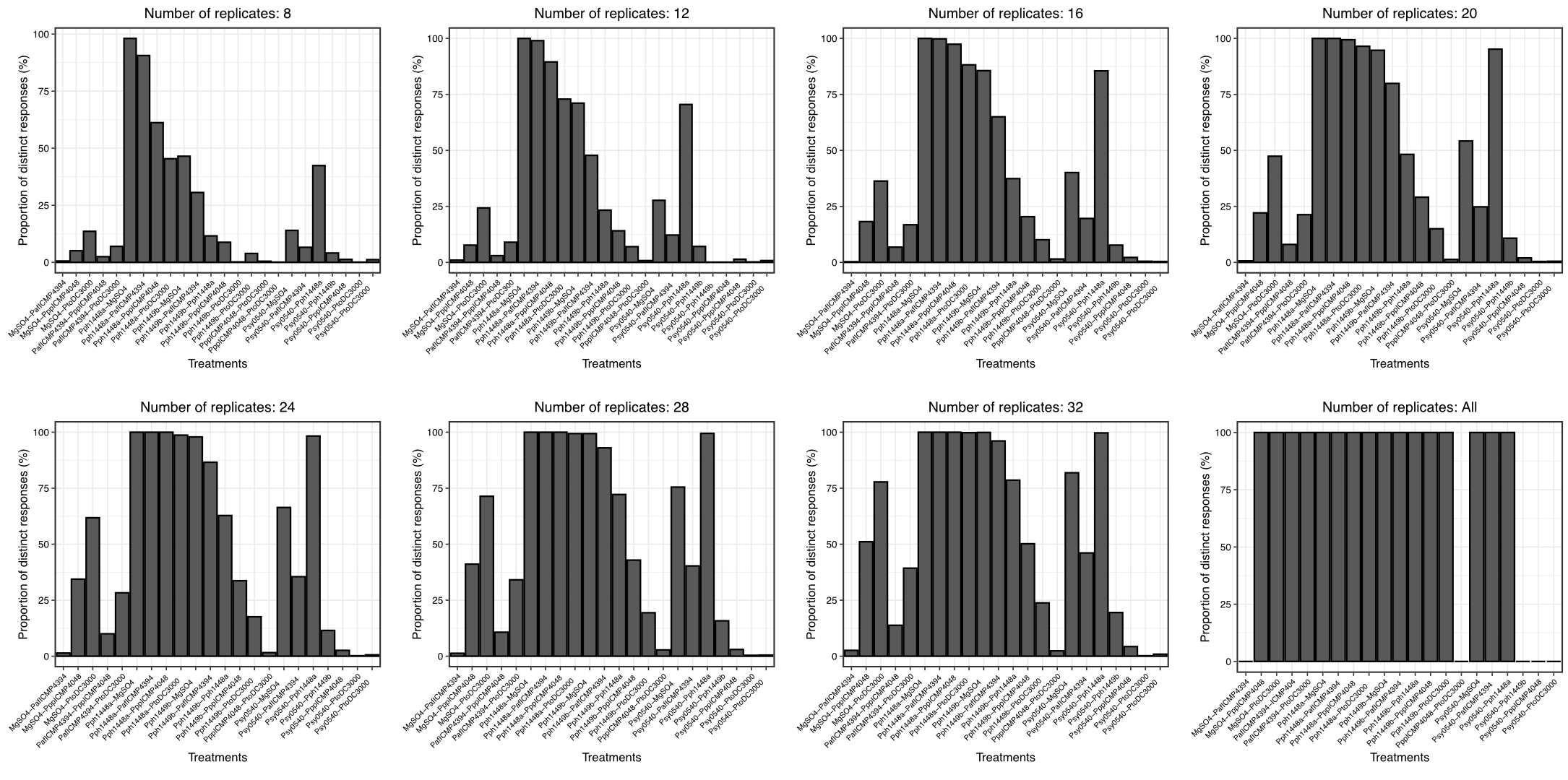

Supplement: S3 Fig — Bean plants were seed infected with 6 P. syringae isolates with a high number of replicates (>50). Plant weights were randomly selected according to various replicate sizes (8–32). Our ability to distinguish pathogens from non-pathogens using Tukey-HSD tests plateaus at 20 replicates per treatment. (TIF) [file ppat.1010716.s003.tif]

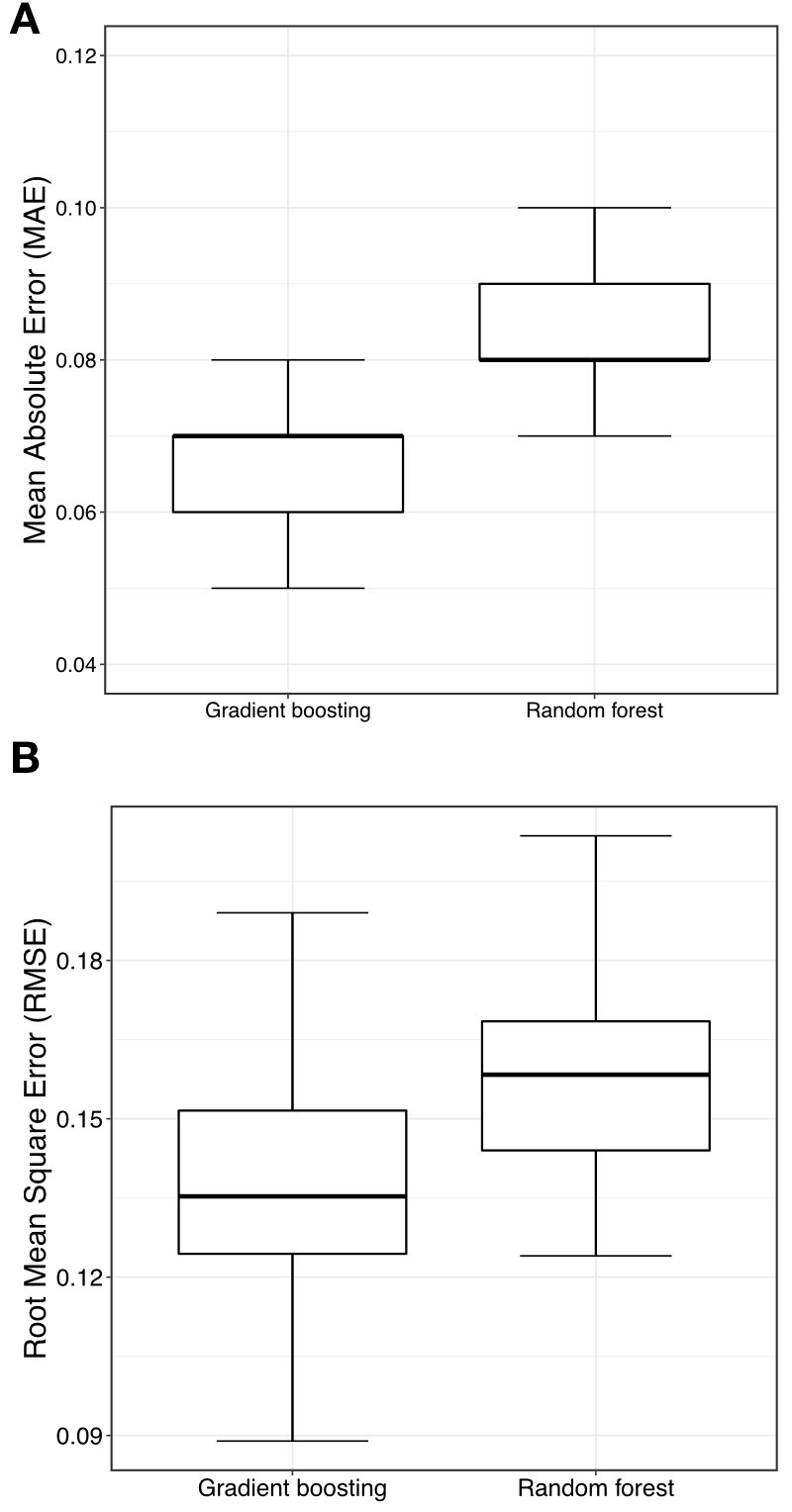

Supplement: S4 Fig — (A) Mean Absolute Error (MAE) as a function of number of samples across 50 cross-validation splits. (B) Root Mean Square Error (RMSE) as a function of number of samples across 50 cross-validation splits. (TIF) [file ppat.1010716.s004.tif]

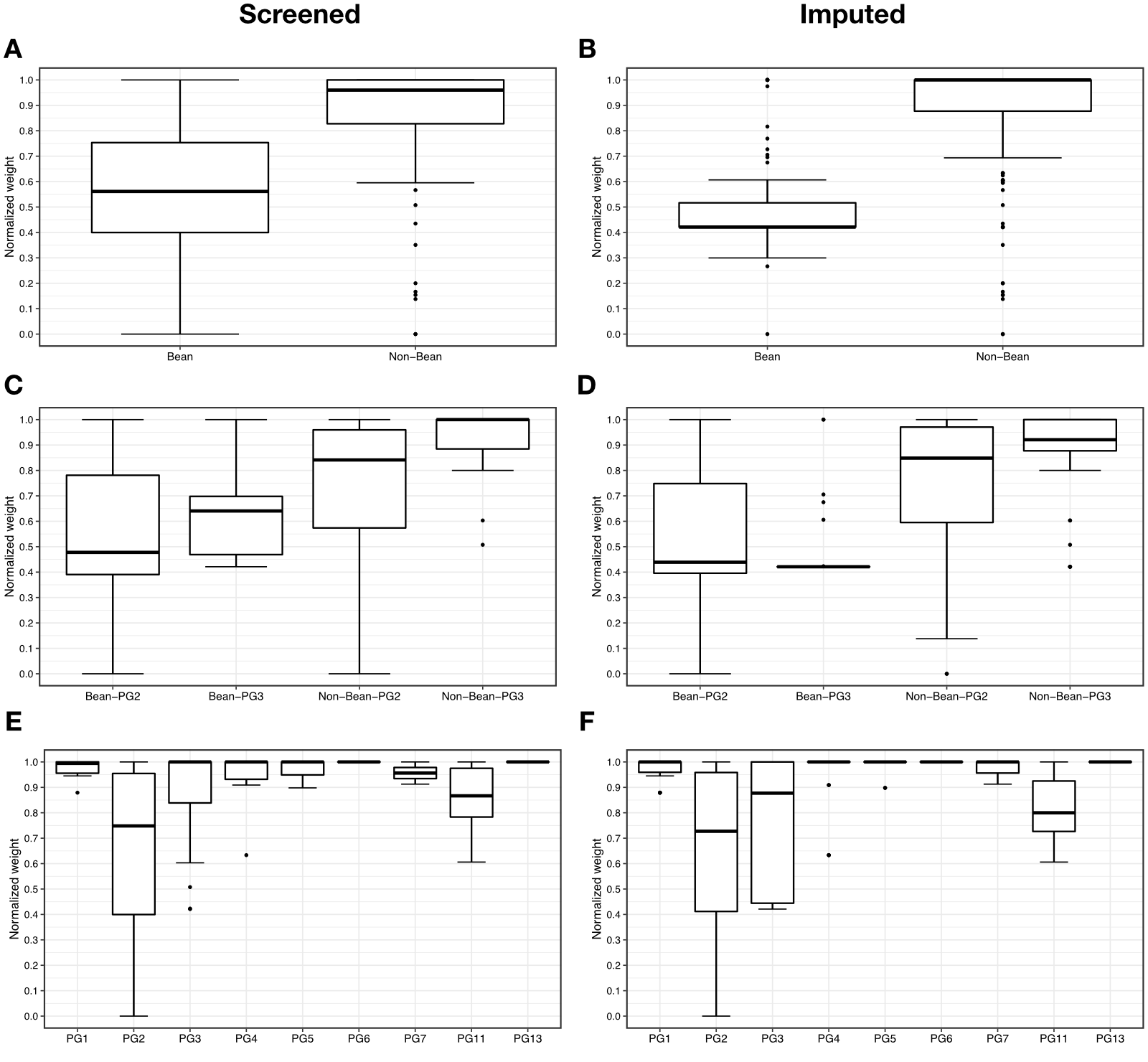

Supplement: S5 Fig — Boxplots showing the distribution of virulence (i.e., normalized plant weight 14 days after seed infection) values for (A) bean verses non-bean isolates for the set of 121 screened strains, and (B) for the 320 strains in the expanded dataset that includes both screened and imputed strains. (C) Distribution of virulence values for bean and non-bean isolates stratified by phylogroup for the screened strains and (D) for the expanded dataset. (E) Distribution of virulence values stratified by phylogroup (PG) for the screened strains, and the (F) expanded strain set. (TIF) [file ppat.1010716.s005.tif]

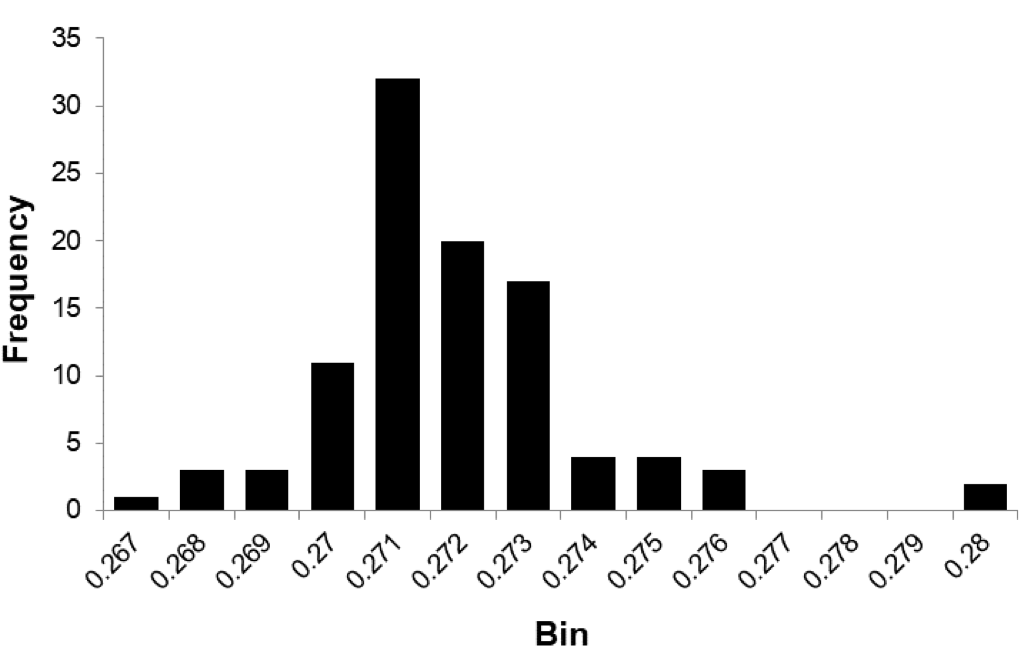

Supplement: S6 Fig — Models were trained with whole-genome k-mers on the expanded strain collection by randomly swapping host of isolation labels. The average of the permuted distribution is 0.271±0.002 (sd). The observed RMSE using the equivalent model design was 0.140. (TIF) [file ppat.1010716.s006.tif]

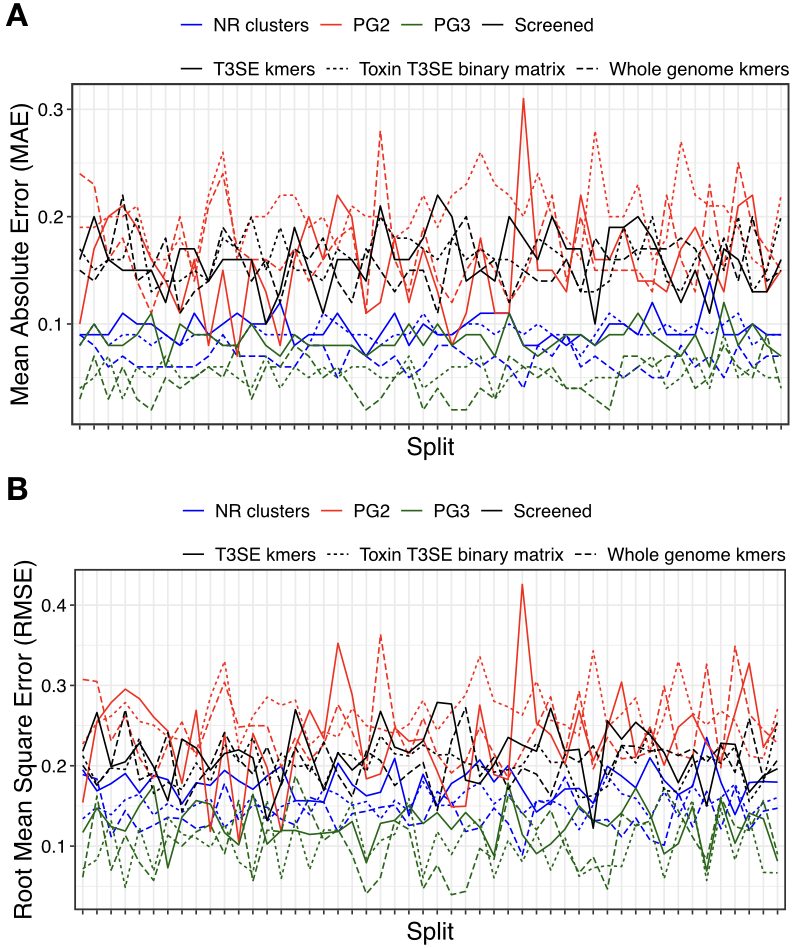

Supplement: S7 Fig — Model performance in terms of (A) MAE and (B) RMSE. Plant weight distributions were kept the same across splits. (TIF) [file ppat.1010716.s007.tif]
